# Human Activity Classification Using Multilayer Perceptron

**DOI:** 10.3390/s21186207

**Published:** 2021-09-16

**Authors:** Ojan Majidzadeh Gorjani, Radek Byrtus, Jakub Dohnal, Petr Bilik, Jiri Koziorek, Radek Martinek

**Affiliations:** Department of Cybernetics and Biomedical Engineering, Faculty of Electrical Engineering and Computer Science, VSB—Technical University of Ostrava, 700 30 Ostrava, Czech Republic; radek.byrtus@vsb.cz (R.B.); jakub.dohnal@vsb.cz (J.D.); petr.bilik@vsb.cz (P.B.); jiri.koziorek@vsb.cz (J.K.); radek.martinek@vsb.cz (R.M.)

**Keywords:** human activity recognition, artificial neural network (ANN), intelligent buildings (IB), smart home (SH)

## Abstract

The number of smart homes is rapidly increasing. Smart homes typically feature functions such as voice-activated functions, automation, monitoring, and tracking events. Besides comfort and convenience, the integration of smart home functionality with data processing methods can provide valuable information about the well-being of the smart home residence. This study is aimed at taking the data analysis within smart homes beyond occupancy monitoring and fall detection. This work uses a multilayer perceptron neural network to recognize multiple human activities from wrist- and ankle-worn devices. The developed models show very high recognition accuracy across all activity classes. The cross-validation results indicate accuracy levels above 98% across all models, and scoring evaluation methods only resulted in an average accuracy reduction of 10%.

## 1. Introduction

The availability and affordability of smart home technology have driven the rapid increase in the number of smart homes. Typically, smart home technologies enable voice-activated functions, automation, monitoring, and tracking events such as the status of windows and doors, entry, and presence detection. Besides comfort and convenience, the integration of smart home functionality with the Internet of Things (IoT) and other communications systems creates new possibilities for assisting and monitoring the well-being of aged or disabled people [1]. In particular, activity recognition within smart homes can provide valuable information about the well-being of the smart home residence. Such information can be utilized to automatically adjust the ambient conditions of the rooms with the use of heating, ventilation, and air conditioning (HVAC). Another use of this information could be the detection of irregularities within the residence’s activities that indicate that assistance is required or a medical emergency. In general, human activity recognition systems can be applied to many fields, such as assisted living, injury detection, personal healthcare, elderly care, fall detection, rehabilitation, entertainment, and surveillance in smart home environments [2].

In general, human activity recognition is formulated as a classification problem. It is an important research topic in pattern recognition and pervasive computing [3]. A significant amount of literature concerning machine learning techniques has focused on the automatic recognition of activities performed by people and the diversity of approaches and methods to address this issue [4,5]. Minarno et al. [6] compared the performance of logistic regression and support vector machine to recognize activities such as lying down, standing, sitting, walking, and walking upstairs or downstairs. Guan et al. [7] tackled this issue using wearable deep LSTM learners for activity recognition. Ramamurthy et al. [8] noted that deep learning methods applied to human activity recognition commonly represent the data better compared to the handcrafted features, due to their advantage of hierarchically self-derived features. Jiang et al. [9] proposed using accelerometer data and convolutional neural networks for real-time human activity recognition. Lee et al. [10] also considered using accelerometer data and a convolutional neural network and obtained 92.71% recognition accuracy. Wan et al. [11] compared four algorithms of neutral networks (convolutional, long short-term memory, bidirectional long short-term memory, multilayer perceptron) in the recognition of human behavior from smartphone accelerometer data. Murad et al. [12] noted that the size of convolutional kernels restricts the captured range of dependencies between data samples and suggested using deep recurrent neural networks instead.

This work proposes the use of two body-worn devices worn on the wrist and ankle. These devices measure temperature, humidity, proximity, magnetic field, acceleration, and rotation and transmit live data to a local host computer. Based on the received data and the use of artificial neural networks, the local host computer can recognize few human activity classes. In our previous works [13], IBM SPSS Modeler and IBM SPSS statistics were used to implement feed-forward neural networks and logistical regression. IBM SPSS Modeler and IBM SPSS statistics are software tools that are commonly used to implement statistical methods. The developed models were designed to recognize multiple pre-defined human activities. Overall, the models showed acceptable levels of recognition accuracy. However, a few shortcomings need to be addressed; for example, some activity categories were too general and difficult to predict, only one test subject was used in the experiment, using two different measurement systems caused synchronization problems, and the accuracy differences between cross-validation and scoring results showed that larger datasets are required. This work aims to solve the problems related to the mentioned issues by using a new methodology. Since the previous works clearly showed the superiority of neural networks, this work utilizes a multilayer perception neural network. For simplicity of measurement and to address data synchronization issues, the use of room ambient data has been eliminated. Besides introducing new activity classes, the least consistent activity classes have been replaced with more specific activity classes, which results in better recognition accuracy. To increase the measurement data size, multiple test subjects were used and new types of equipment were utilized to increase the sampling rate. The above changes resulted in significant recognition accuracy improvements. Overall, this work aimed to increase the recognition accuracy and the number of recognizable activities, and to provide a practical solution that eliminates the typical computational limitations of wearable devices.

## 2. Related Works

In recent years, the data analysis within smart homes has gained significant attention among researchers. Geraldo et al. [14] proposed an intelligent decision-making system for a residential distributed automation infrastructure based on wireless sensors and actuators. The method increased the precision in decision-making with a neural network model and reduced node energy consumption using a temporal correlation mechanism. Ueyama et al. [15] used a probabilistic technique for monitoring a remote alert system for energy. A Markov chain model was used to calculate the entropy of each device monitored, and the method identified novelties with the use of a machine learning algorithm. The results showed that the method could reduce the power consumption of the monitored equipment by 13.7%. Rocha et al. [16] proposed an intelligent decision system based on the fog computing paradigm, which provides efficient management of residential applications. The proposed solution was evaluated both in simulated and real environments. Goncalves et al. [17] determined and mapped out the physical and emotional state of home care users, implemented a participatory design that included the user within its social, psychological, and therapeutic context, and explored the flexible method when applied to older users. Subbaraj et al. [18] described the process of checking the consistent behavior of a context-aware system in a smart home environment using formal modeling and verification methods. The results confirmed the consistent behavior of the context-aware system in the smart environment. Torres et al. [19] designed an offloading algorithm to ensure resource provision in a microfog and synchronize the complexity of data processing through a healthcare environment architecture, and they experimented with face recognition and fall detection. Balakrishnan et al. [20] discussed and reviewed the literature on the smart home definition, purpose, benefits, and technologies. Tax et al. [21] investigated the performance of several techniques for human behavior prediction in a smart home. Azzi et al. [22] proposed to use a very fast decision tree for activity recognition and formulated activity recognition as a classification problem where classes correspond to activities. Sim et al. [23] proposed an acoustic information-based behavior detection algorithm for use in private spaces. The system classified human activities using acoustic information, combined strategies of elimination and similarity, and established new rules.

Much of the research in the indirect activity recognition field is focused on fall detection [24,25]. Sadreazami et al. [24] utilized Standoff Radar and a time series-based method to detect fall incidents. Ahamed et al. [25] used accelerometer-based data and deep learning methods for fall detection. Other researchers took activity recognition further than fall detection by recognizing multiple human behaviors. Commonly, camera-based recognition techniques are used to recognize multiple predefined human activities. Hsueh et al. [26] used deep learning techniques to learn the long-term dependencies in a multi-view detection framework to recognize human behavior. Besides the computational burden, the camera-based solutions frequently introduce privacy and security concerns for the residence. Therefore, indirect recognition methods are generally preferred. Indirect recognition methods are often limited to presence detection and occupancy monitoring. Szczurek et al. [27] investigated occupancy determination based on time series of $CO_{2}$ concentration, temperature, and relative humidity. Vanus et al. [28] designed a $CO_{2}$-based method for human presence monitoring in an intelligent building. The work continued by replacing measured $CO_{2}$ with predicted values of $CO_{2}$. Predictions were performed on neural networks [29], random trees, and linear regression [30].

On a larger scale, others have taken indirect recognition to a more advanced level by recognizing specific human activities. Kasteren et al. [31] introduced a sensor and annotation system for performing activity recognition in a house setting using a hidden Markov model and conditional random fields, resulting in class accuracy of 79.4%. Nweke et al. [2] reviewed deep learning algorithms for human activity recognition using mobile and wearable sensor networks. Albert et al. [32] used mobile phones for activity recognition in Parkinson’s patients. Hassan et al. [33] proposed using smartphone inertial sensors such as accelerometers and gyroscope sensors to recognize human activities. The obtained results showed a mean recognition rate of 89.61%. Zhou et al. [34] used deep learning and datasets collected from smartphones and on-body wearable devices to perform human activity recognition within the Internet of Healthcare Things. In similar studies, Kwapisz et al. [35] and Bayat et al. [36] also suggested using smartphones.

The use of a smartphone as the primary sensor is very convenient but it comes with major drawbacks. In practice, they fail to identify complicated and real-time human activities. Ravi et al. [37] found that using a single triaxial accelerometer to recognize human activity can result in fairly accurate results. The work showed the limitation of a single worn sensor near the pelvic region when it comes to activities that involve the movement of only the hands or mouth. Chen et al. [38] noted the variety of smartphone positions or orientations, and the gross accuracy of their embedded sensors could result in additional challenges. Other works investigated the use of multiple sensors. Bao et al.’s [39] implementation involved five small biaxial accelerometers worn simultaneously on different parts of the body; decision tree classifiers showed an overall accuracy rate of 84%. Furthermore, the research showed that the recognition accuracy only drops slightly when only two thigh- and wrist-worn sensors are used. Trost et al. [40] compared results obtained from hip- and wrist-worn accelerometer data for the recognition of seven classes of activities. On the other hand, Zhang et al. [41] noted that the computational limitations of wearable devices can also represent a challenge in real-world applications. Our implementation involves wrist-worn and ankle-worn devices that communicate wirelessly with a remote computer, which eliminates computational limitations. These limitations have been eliminated by the use of a powerful local host computer.

## 3. Methods

The proposed method consists of three main stages: data acquisition, pre-processing, and predictive analytics. Two individual wearable gadgets based on STMicroelectronics development boards are used to record the movements of the test subjects. With the use of wireless technology, the obtained information is directly sent to a local host computer. After data buffering and synchronization, the local host computer performs human activity recognition using artificial neural networks. The recognition result may be sent to online cloud services for remote monitoring and visualization. Table 1 shows a list of the nine activity classes that were used in this research. These classes represent a few of the most performed daily human activities. This section describes the measurements, data acquisition methods, and applied mathematical models.

### 3.1. Measurements and Data Acquisition Methods

**Figure 1:**
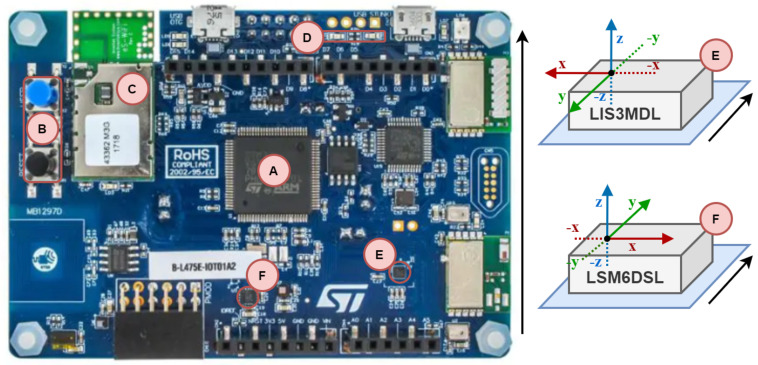
The printed board of B-L475E-IOT02A development board. (A: MCU, B: User button, C: Wifi module, D: LEDs, E: 3D Magnetometer, F: 3D Accelerometer and Gyroscope).

A development board, B-L475E-IOT01A2 from STMicroelectronics, was used for data acquisition [42]. It is based on an ultra-low-power MCU from STM32L4 series with other modules for communication (Bluetooth, Wi-Fi, Sub-RF, NFC) and embedded Micro-Electro-Mechanical Systems (MEMS) sensors for monitoring environmental parameters (temperature, humidity, proximity, magnetic field) and mechanical quantities (acceleration, rotation) [43]. Thanks to its concept and low cost, the development board enables fast design and commissioning. Figure 1 shows a detailed diagram of the components of the development board used for measurement and data acquisition. Microcontroller unit (MCU) “A” collects and processes data from onboard sensors (“F” and “E”). The received data are then processed and prepared into a data structure to be sent to the Transmission Control Protocol (TCP) server. Sending takes place asynchronously via the WiFi module (C). This means that sending is initiated, for example, every 50 ms, independently of the main program cycle. The current program status is indicated by onboard LEDs (D) and can be modified via a user button (B).

**Figure 2:**
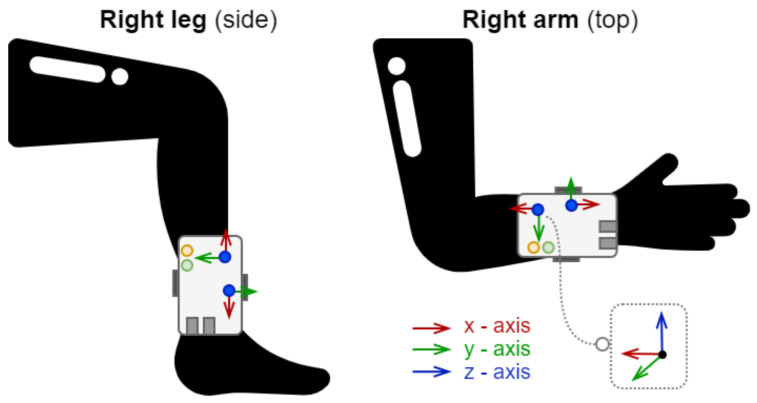
Location of wearable measurements devices on the body and orientation of the sensors.

The designed measurement chain, for the purpose of recording human movement, consists of a high-performance 3-axis magnetometer (E, LIS3MDL), 3D accelerometer, and 3D gyroscope (F, LSM6DSL) [44,45]. These sensors allow the recording of 3-axis acceleration up to ±16 g and 3-axis angular rate up to ±2000 dps with up to 1 kHz readout frequency; they also record the 3-axis strength of the magnetic field and orientation up to ±16 gauss with up to 80 Hz readout frequency. The actual orientation of the sensors is demonstrated in Figure 2.

**Figure 3:**
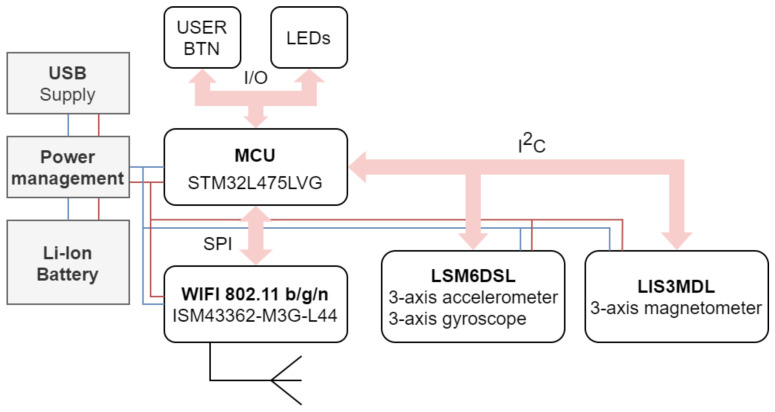
Hardware and communication architecture.

Figure 3 shows the utilized hardware and communication architecture of the data acquisition stage. Data are sent via a Wi-Fi module from Inventek Systems (C, ISM43362-M3G-L44) using the 802.11n protocol. The Inter-Integrated Circuit ($I^{2}C$) bus is used for communication with the sensors and the Serial Peripheral Interface (SPI) bus for communication with the Wi-Fi module. To guarantee sufficient mobility, the devices are powered by a Li-Ion-based power source. The data chain consists of two wireless measurement modules (TCP clients based on B-L475E-IOT01A2) and a remote personal computer. The personal computer is also used as a Wi-Fi Access Point (AP) for TCP clients. Communication between these two sides is based on the TCP/IP protocol, where static IPv4 addressing is used. This solution allows recording of the movement of a person within the distance of 25 m in open space (actual distance depends on type and number of obstacles within the path of the signal) from the position of the host computer. Additionally, this solution provides sufficient communication speed and bandwidth for data transfer between all participating devices.

**Figure 4:**
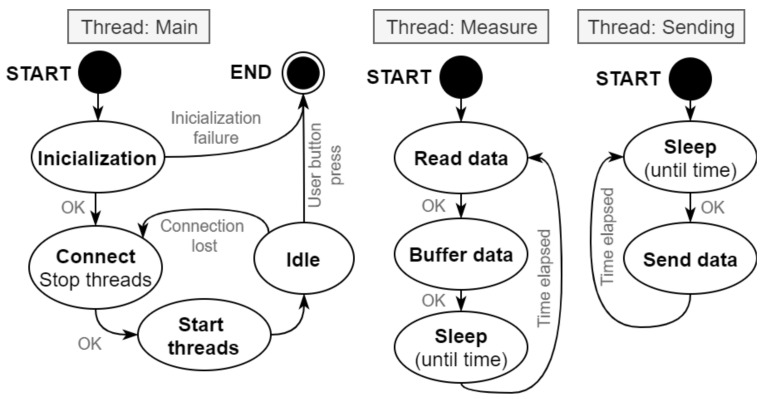
State diagram of the proposed measurement system.

As Figure 4 illustrates, the onboard system initializes necessary modules. If the initialization is successful, the system establishes a TCP connection and starts two other threads. The measurement thread is used for data acquisition. After measuring all required quantities using sensors, the thread prepares the data for sending. The prepared data row is moved to the data buffer for sending and then sent to the TCP server for further processing. Sending of data is processed in another thread. The program in this thread is processed every 15 ms. Proper timing and synchronization of the measured quantities is also necessary. This is guaranteed by the Real-Time Operational System (RTOS) with the implementation of the software [46]. The sampling rate is programmatically set to 66.6 Hz, which results in a single record every 15 ms.

**Figure 5:**
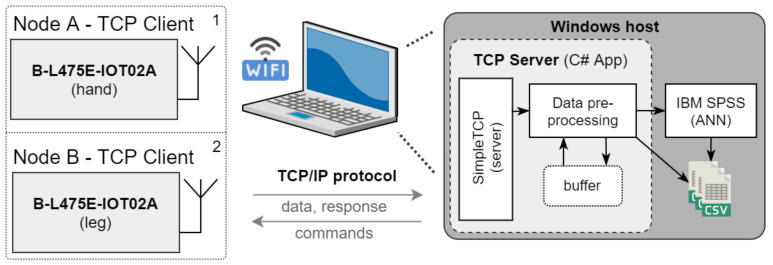
Data transfer chain of the measurement system.

For the data to be properly transferred from the client to the server, a connection must be established. Clients register before sending data on the server-side. After this, data can be transferred. Within the TCP server, explained in Figure 5, the data are pre-processed and stored in files. Due to the possibility of connection loss between the communicating devices, the TCP server is equipped with a data buffer. The buffer is used as a form of data cache in case of data transfer failure of one of the clients. The TCP server then receives more data at once. This is also necessary for preventing data inconsistency. In the buffer, up to 50 data rows from each TCP client can be saved. There is also an implementation to discard old irrelevant samples. The TCP server application is written in C#. It consists of the SimpleTCP library and its class is called Saver [47]. The SimpleTCP server registers all clients and receives data from them. Using a received event, the data are passed to the pre-processing routine, where data are prepared for saving and use in IBM SPSS. After they are prepared, data are moved into the buffer and synchronized data samples are finally saved.

### 3.2. Recognition Using Artificial Neural Networks

**Figure 6:**
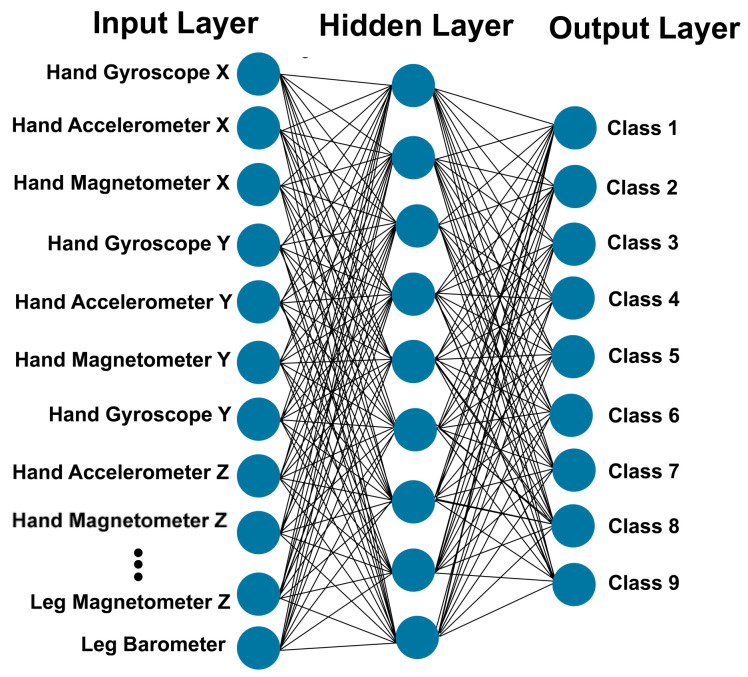
Example of applied neural network with 9 neurons at the hidden layer.

This section describes the statistical and mathematical methods used for recognition and evaluation. Using known examples (training sets) to estimate a function is known as supervised learning. Supervised learning in artificial neural networks is aimed at the estimation of underlying functions [48,49,50,51]. Artificial neural networks are very popular for modeling non-linear problems and for the prediction of the output values for given input parameters from their training values. Iwendi et al. [52] used recurrent neural networks for cyberbullying detection. Sun et al. [53] developed a neural network solution to evaluate the risk of credit card delinquency based on the spending behaviors and the client’s personal characteristics. Pinardi et al. [54] explored the application of neural networks in atmospheric river forecasting. Multilayer perceptron (MLP) is one of the most commonly used types of artificial neural networks; it utilizes backpropagation for training (a supervised learning technique). The standard architecture of an MLP artificial neural network consists of an input layer, multiple hidden layers, and an output layer. The input layer dedicates an independent input neuron to each input variable with one. The hidden layer contains the core logic of the network. The output layer provides the predicted values. Figure 6 shows an example of an MLP network with a single hidden layer.

The MLP artificial neural network can be mathematically described as follows [55]:

Input layer: $j_{o}=p\;\;\;units,a_{0:1},\ldots ,a_{0:j_{0}};$ with

(1) $$a_{0:j_{0}}=x_{j},$$

where *j* is the number of neurons in the layer and *X* is the input.

$i^{th}$ hidden layer: $j_{i}\;\;\;units,a_{i:1},\ldots ,a_{i:j_{i}};$ with

(2) $$a_{i:k}=\gamma_{i}\left(C_{i:k}\right)$$

and

(3) $$C_{i:k}=\sum_{j=0}^{J_{i}-1}\omega_{I:j_{1},k}a_{i-1:j},$$

where $a_{i-1:0}=1$, $\omega_{I:j_{1},k}$ is the weight leading from layer i−1, unit *j* to layer *i*, unit *k*. $\gamma_{i}$ is the hyperbolic tangent activation function for layer *i* and it is described as follows.

(4) $$\gamma_{\left(c_{k}\right)}=tanh\left(c\right)\frac{e^{c}-e^{-c}}{e^{c}+e^{-c}}$$

where $a_{i-1:0}=1$.

Output layer: $j_{I}=R\;\;\;units,a_{I:1},\ldots ,a_{I:J_{I}};$ with

(5) $$a_{I:k}=\gamma_{I}\left(C_{I:k}\right)$$

and

(6) $$C_{I:k}=\sum_{j=0}^{J_{1}}\omega_{I:j_{1},k}a_{i-1:j},$$

where $a_{i-1:0}=1$.

The activation function of the output layer defines how the weighted sum of the input is transformed; the softMax function is used as an activation function for the output layer.

(7) $$\gamma_{\left(c_{k}\right)}=\frac{e^{c_{k}}}{\sum_{j\in \Gamma_{h}}e^{c_{j}}}$$

Stopping rules determine when to stop MLP training. Training proceeds through at least one cycle, and then it can be stopped according to one of the criteria in Table 2. To avoid excessive future training duration, it is better to select results with SR1 and SR2 criteria.

The training accuracy is not sufficient to estimate the response of the trained MLP networks to unknown future input. Cross-valuation is the most commonly used method to estimate the true performance of statistical methods. If data are not scarce, the dataset is split into three segments for training, testing, validation. This splitting can be performed using multiple different method, and computing an average score over different partitions can reduce bias [56,57,58]. The models are trained using the training partition and evaluated using the testing and validation partitions. Most researchers only rely on the validation results and skip the scoring stage. In the scoring stage, the models are trained and validated using entirely different datasets. Typically, cross-validation demonstrates the accuracy of models for a very large dataset, while scoring shows the real accuracy of the model with the current training dataset.

## 4. Measurements and Results

The data acquisition was performed in laboratory EB412 at the new Faculty of Electrical Engineering and Computer Science building of the VSB Technical University of Ostrava. Six datasets were obtained as the results of these measurements. Table 3 shows the number records in each recorded dataset, where individual letters are assigned to different test subjects and numbers represent different measurement dates. This section evaluates the recognition accuracy of the developed models with the use of cross-validation and scoring.

**Figure 7:**
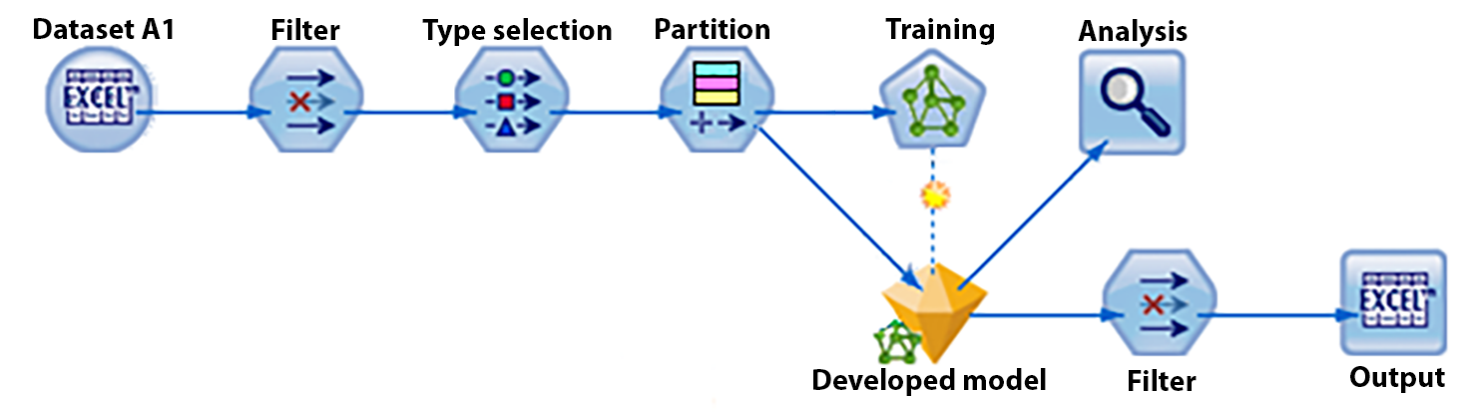
Validation data stream developed in IBM SPSS.

The analysis was performed using IBM SPSS Modeler. In the first stage, models were trained and evaluated using cross-validation. Figure 7 shows the developed data stream. It starts with importing the data and continues with selecting relevant data and assigning a specific type to each datum. Once the input data are established, the partition nodes split the data into three subsets: training (30% of total data), testing (30% of total data), validation (40% of total data). In the next stage, an MLP network is trained, tested, and validated using the above partitions.

The above steps were repeated for seven model settings and six different datasets, which resulted in 42 models. These models mostly showed accuracy levels above 99%, which is considerably more accurate than similar implementations. A minimum accuracy of 94.59% was observed in dataset B1, activity class 1, with eight neurons in the hidden layer. On other hand, many models showed 100% accuracy across multiple activity classes and neuron settings. Table 4 shows the average accuracy of the models across all nine classes. In general, it can be observed that an increase in the number of neurons slightly improves the accuracy, but this accuracy improvement reverses in models with more than 128 hidden layer neurons. A closer look shows that these models are limited by the maximum allowed training time (stopping rule SR3). Therefore, the lowest possible error state and highest accuracy cannot be reached by these models.

Table 5 represents the average accuracy of each activity class across multiple datasets. Class 4 is the most accurate and often shows 100% recognition accuracy on average. Given that it corresponds to relaxing and minimal movement, this is a very consistent activity and is easy to recognize. All other activity classes maintained average accuracy levels above 98.86%.

**Figure 8:**
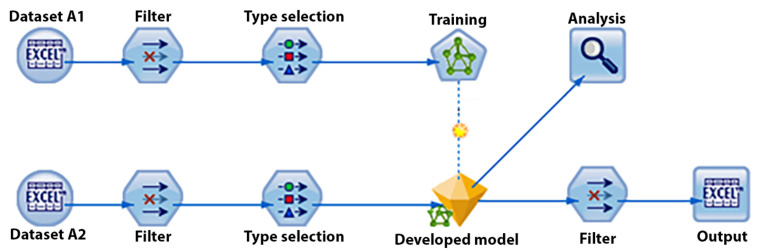
Scoring data stream developed in IBM SPSS.

The above results demonstrate extremely accurate recognition rates and the high potential of the introduced method. In general, the training dataset and validation dataset are very similar in cross-validation. Therefore, this indicates the accuracy of models that are trained with a very large training dataset that includes most of the possible events. Often, most researchers only rely on these cross-validation results. However, to estimate the real performance of the models for certain datasets, it is recommended to use an entirely different dataset for training and evaluation. This process is called scoring. Figure 8 shows a scoring data stream. Dataset A1 is entirely used for training, and dataset A2 is only used for evaluation. Since the scored models have never observed the evaluation datasets, it is expected that noticeable differences will be observed in the accuracy levels in comparison with the cross-validation results. The larger the difference, the better the indication of the larger training dataset requirement.

Table 6 shows the average scoring accuracy of the models. Scoring dataset A1 and dataset A2 against each other resulted in an average of 91.35% and 91.04%, which is impressive. On the other hand, datasets B1 and B2 experienced a more significant drop (average of 79.45% and 77.45%). Further investigation showed that these significant accuracy drops were only present fpr class 7 and class 9 activities, which is the direct result of the inconsistent actions of the test subject during these activities. Scoring datasets C1 and C2 against each other resulted in 88.72% and 93.60%, which is also an impressive outcome.

Table 7 shows the average scoring accuracy of each activity class across multiple models and datasets. In general, classes 1, 2, 4, and 5 show highly accurate scoring results. On the other hand, class 7’s average accuracy suffers from significant accuracy loss. A closer look shows that this accuracy loss is mainly present in the experiment using datasets B1 and B2. Otherwise, other datasets performed decently across all classes and models. In total, the validation accuracy averaged 99.40% and the scoring accuracy averaged 86.94%. This difference was smaller for specific model settings. Further observations of both evaluations showed that the accuracy levels increased with an increase in the hidden layer neuron count. Typically, this relation reversed after 128 or 256 neurons due to the maximum allowed training times. The model setting with 256 neurons was selected to be the most suitable model setting. The average validation and scoring accuracies of these models were 99.78% and 89.27%, respectively, which shows approximately a 10% difference. This is a significant improvement over previous implementations.

## 5. Discussion

This study aimed to introduce a methodology that addresses most of the concerns within activity recognition research. The initial research showed that using ankle- and wrist-worn wearable devices is optimal in terms of the recognizable number of activities. Using wireless technology to transmit measured body movements and remote processing of data reduces the computational burden on measurement devices. Essentially, this allows simpler and perhaps much smaller devices to be utilized in the future. The remote processing using a powerful local computer also alleviated most of the concerns about the computational limitations of wearable devices and smartphones. This implementation used an MLP with only a single hidden layer, which represents a simpler model and less computationally intensive training. This allows better training of larger models in a given time. With direct comparison with our previous study [13], which used two hidden layers, the cross-validation accuracy was almost identical (within margins of error). However, due to higher and more stable data acquisition rates, the scoring accuracy was significantly improved.

In addition, this study increased the number of recognizable activities to nine. A total of 84 models were developed to examine the recognition accuracy of these activity classes. The models used for cross-validation (42 models) mostly showed accuracy levels above 99%, which is considerably more accurate than similar implementations and our previous study [13]. The relaxing activity showed mostly 100% recognition accuracy levels, and other activities cross-validated to accuracy levels above 98.86%. A minimum accuracy of 94.59% was observed in dataset B1, activity class 1, with eight neurons in the hidden layer. This was expected since dataset B1 represents the smallest data size (Table 3). On the other hand, many models resulted in 100% accuracy across multiple activity classes and neuron settings. According to Table 4, increasing the number of neurons slightly improved the accuracy, but this effect was reversed in larger models due to exceeding the maximum allowed training time threshold, which did not allow the models to reach a minimum recognition error state. The methodology was further tested using the scoring technique, which resulted in additional 42 models (Table 6 and Table 7). As mentioned earlier, all activities showed highly accurate scoring results, but the vacuum cleaning activity’s (class 7) average accuracy suffered from an accuracy loss. Scoring the dataset A1 and dataset A2 against each other resulted in an average of 91.35% and 91.04%, and scoring C1 and C2 against each other resulted in 88.72% and 93.60%, but datasets B1 and B2 experienced a more significant drop (average of 79.45% and 77.45%), which was mainly caused by class 7’s recognition accuracy. Since this problem only exists in one dataset, it can be ruled out as a measurement error. By removing the class 7 results when datasets B1 and B2 were scored against each other, the scoring accuracy was almost on par with the validation results. This shows that a sufficient amount of training data were used in this research. Further observations of both evaluations showed that the accuracy levels increased with an increase in the hidden layer neuron count. Typically, this relation reversed after 128 or 256 neurons due to the maximum allowed training times. Overall, the obtained results demonstrated extremely accurate recognition and the high potential of the introduced method.

This work was aimed at introducing a methodology with high recognition accuracy and without the typical computational limitations that are described in most existing research. The novel measurement methodology of this work addressed many previous concerns, such as inaccurate predefined activity classes, the use of a single test subject, and the utilization of two different measurement systems. In future works, the obtained accuracy levels can be further improved by the use of filters and data buffering to eliminate outliers within the prediction results. In addition, the number of activity classes could be further increased.

## 6. Conclusions

This work addresses many previous concerns. These issues are resolved by the use of a new methodology. It utilizes a multi-layer perceptron neural network and a novel data acquisition method to recognize nine different human activity classes, with impressive accuracy levels. The developed models cross-validated to accuracy levels above 98% across all activity classes. Thanks to the use of higher data acquisition rates and subsequently larger datasets, the accuracy difference between cross-validation and scoring was reduced to only 10%. Overall, the recognition accuracy levels were noticeably improved in comparison with the previous implementation. However, allowing longer training times may increase the accuracy levels in larger neural networks and allow even more accurate results. In addition, these results may be further improved by the use of filters and data buffering to eliminate outliers within the prediction results. The novelty of this work lies within the simplified recognition methods, highly accurate recognition accuracy levels, elimination of the computational burden by the use of a remote computer, variety of recognizable activities, and the possibilities of integration with smart home technologies. In future works, the trained models will be used in a real-time system that allows live recognition of the smart home residence, with integration and communication with smart home technologies and IoT (Internet of Things) platforms. Furthermore, the number of test subjects, recognizable activities, and accuracy levels will be increased.

## Figures and Tables

**Table 1:** The list and description of the measured activities.

Activity Class	Description
Class 1	Climbing down the stairs
Class 2	Climbing up the stairs
Class 3	Using a computer
Class 4	Relaxing
Class 5	Running
Class 6	Standing
Class 7	Vacuum cleaning
Class 8	Walking
Class 9	Writing using a pen

**Table 2:** List and description of the stopping rules.

Stopping Rule	Description
SR1	Minimum relative change in error achieved
SR2	Error cannot be further decreased
SR3	Maximum training time has been exceeded

**Table 3:** Datasets size.

Dataset	Size in Terms of Number of Records (-)
A1	128,300
A2	202,506
B1	114,560
B2	165,224
C1	135,451
C2	181,023

**Table 4:** Cross-validation accuracy of the developed model.

Neuron Count	Dataset	SR	Accuracy (%)	Dataset	SR	Accuracy (%)
8	A1	SR1	98.10	A2	SR2	98.60
16	A1	SR2	99.40	A2	SR1	99.30
32	A1	SR2	99.70	A2	SR2	99.70
64	A1	SR2	99.80	A2	SR2	99.80
128	A1	SR2	99.90	A2	SR3	99.90
256	A1	SR3	99.90	A2	SR3	99.80
512	A1	SR3	99.70	A2	SR3	99.60
8	B1	SR2	98.60	B2	SR2	99.70
16	B1	SR2	99.30	B2	SR2	98.90
32	B1	SR2	99.50	B2	SR2	99.50
64	B1	SR2	99.60	B2	SR2	99.70
128	B1	SR2	99.70	B2	SR2	99.80
256	B1	SR2	99.70	B2	SR3	99.90
512	B1	SR3	99.50	B2	SR3	99.70
8	C1	SR2	98.00	C2	SR1	98.48
16	C1	SR2	98.90	C2	SR2	99.16
32	C1	SR2	99.20	C2	SR2	99.50
64	C1	SR2	99.50	C2	SR2	99.67
128	C1	SR2	99.70	C2	SR2	99.77
256	C1	SR3	99.60	C2	SR3	99.77
512	C1	SR3	99.40	C2	SR3	99.60

**Table 5:** Average cross-validation activity classes accuracy.

Activity Class	A1 (%)	A2 (%)	B1 (%)	B2 (%)	C1 (%)	C2 (%)	Average (%)
1	99.26	98.87	99.04	98.88	99.00	98.14	98.86
2	99.53	99.41	98.96	99.20	99.20	98.85	99.19
3	99.99	99.94	99.97	99.94	99.98	99.82	99.94
4	100.00	99.97	99.99	99.99	100.00	99.99	99.99
5	99.28	99.32	99.49	99.36	98.72	99.46	99.27
6	99.63	99.49	99.64	99.54	99.68	99.68	99.61
7	99.15	98.97	99.96	99.97	98.72	98.44	99.20
8	98.89	99.08	98.15	98.19	98.24	98.22	98.46
9	99.95	99.95	99.93	99.92	99.93	99.88	99.93

**Table 6:** Scoring accuracy of the developed model.

Neuron Count (-)	A1XA2 (%)	A2XA1 (%)	B1XB2 (%)	B2XB1 (%)	C1XC2 (%)	C2XC1 (%)
8	88.43	92.80	73.07	77.61	88.05	92.52
16	94.91	89.19	80.32	77.39	87.42	93.61
32	89.82	87.83	76.77	78.54	83.37	96.32
64	89.02	95.82	83.73	78.09	89.81	95.23
128	94.04	96.79	78.82	78.52	91.76	93.52
256	93.35	96.05	83.12	77.79	90.90	94.42
512	89.90	78.82	80.28	74.25	89.70	89.59
**Average**	91.35	91.04	79.45	77.45	88.72	93.60

**Table 7:** Average scoring activity classes accuracy.

Activity	A1XA2	A2XA1	B1XB2	B2XB1	C1XC2	C2XC1	Average
Class	(%)	(%)	(%)	(%)	(%)	(%)	(%)
1	95.52	95.15	91.68	93.17	96.77	96.97	94.88
2	94.78	94.55	95.73	95.38	93.87	96.87	95.20
3	77.78	86.96	86.91	94.25	82.43	94.84	87.20
4	99.78	99.26	88.04	84.04	91.86	99.38	93.72
5	98.10	95.22	87.43	87.99	95.93	89.96	92.44
6	92.12	82.48	77.58	88.75	86.76	88.49	86.03
7	91.16	87.85	37.92	7.31	73.24	89.91	64.56
8	93.61	88.05	73.38	57.83	92.14	92.82	82.97
9	79.32	89.83	76.33	88.38	85.48	93.18	85.42

## Data Availability

The data presented in this study are available on request from the corresponding author.
